# No increase of biopsy rates despite high rates of probable eosinophilic esophagitis in patients with esophageal food impaction

**DOI:** 10.1007/s00508-025-02542-6

**Published:** 2025-05-21

**Authors:** Lukas Neumann, Rafael Brader, Lili Kazemi-Shirazi, Michael Trauner, Christian Primas, Karin Wisniowski, Jurij Maurer, Hansjörg Schlager, Jagoda Pokryszka, Clemens Dejaco, Walter Reinisch, Gottfried Novacek, Philipp Schreiner

**Affiliations:** 1 Division of Gastroenterology and Hepatology, Department of Internal Medicine III, Medical University of Vienna, Vienna, Austria; 2 Sigmund Freud University, Vienna, Austria; 3 Division of Gastroenterology and Hepatology, Krankenanstalt Rudolfstiftung, Krankenanstaltenverbund Wien (KAV), Vienna, Austria; 4 Division of Gastroenterology and Hepatology, Department of Internal Medicine, Medical University of Graz, Graz, Austria

**Keywords:** Emergency room, Esophagogastroduodenoscopy, Esophageal disease, Swallowing disorder, Dysphagia

## Abstract

**Introduction:**

Eosinophilic esophagitis (EoE) is one of the main causes of esophageal food impaction (EFI). Since only few endoscopists take biopsies during the emergency endoscopy at EFI presentation, as is recommended by current guidelines, a high number of patients will not have a proper diagnosis after EFI. Hence, we investigated the change of biopsy rates and the etiology of EFI over 11 years.

**Methods:**

All patients presenting at the emergency department (ED) of a tertiary center with an EFI who underwent esophagogastroduodenoscopy (EGD) between 2013 and 2023 were included. Clinical and endoscopic variables were analyzed retrospectively. We performed a binary logistic regression model to predict biopsy performance.

**Results:**

A total of 180 EFI cases (67% male, median age 57 years) were recorded between 2013 and 2023. Overall, esophageal biopsies were taken only in 18% without any increase over time. In patients ≥ 50 years of age (*n* = 108), the etiology remained unknown in half of patients (48%), followed by gastroesophageal reflux disease (GERD; 23%) and EoE (18%). However, in patients < 50 years of age, EoE was the main suspected etiology in 69% of cases. Biopsies were obtained in only 18% of all patients with suspected EoE. Age, gender, and the year of EFI were not associated with biopsy performance. However, the probability of biopsy increased by a factor of 4.03 in the presence of suspected EoE by the endoscopist.

**Conclusion:**

Despite an increasing awareness of EoE, the biopsy rate in EFI is rather low. Routine biopsies should be taken to shorten diagnostic delay.

## Introduction

Eosinophilic esophagitis (EoE) is a chronic immune-mediated disease of the esophagus with an increasing incidence and prevalence in recent decades [1, 2]. The cardinal symptom of adult patients with EoE is dysphagia manifesting in food that becomes stuck or that is travelling slowly down the esophagus. However, since symptoms are often neglected by many patients, first contact with the health system is often at the emergency department (ED) in case of an esophageal food impaction (EFI) [3]. Nowadays, EoE is considered as one of the main causes for EFI in general and the most common reason in patients < 50 years of age [3, 4]. On the other hand, EFI ranks as the third most common nonbiliary emergency encountered in gastroenterology. EFI predominantly affects adults in their fourth or fifth decades of life, although there has been a notable increase in prevalence among young adults, partly attributable to the increasing incidence of EoE. EoE-associated visits to the ED had an even three-fold increase from 2009 to 2019 [5]. However, the number of undiagnosed EoE patients who were having an EFI is supposedly much higher.

Since only histology confirms the diagnosis of EoE, it is of outmost importance to take biopsies during an EFI. Nevertheless, most endoscopists do not take biopsies at the initial event of an EFI resulting in undiagnosed patients [6]. Many of these patients are lost to follow up and will have an undiagnosed EoE as cause of EFI [7, 8, 10]. These shortcomings result in a considerable diagnostic delay with as high as 10 years in one-third of EoE patients [9]. Furthermore, recent research states that failure to identify a potential underlying pathology at index endoscopy is strongly associated with not receiving appropriate postendoscopy care [10]. We therefore aimed to evaluate the biopsy rates and the etiology of EFI over 11 years in a tertiary center in Austria.

## Methods

We assessed the biopsy rate and the etiology of EFI (with and without previously diagnosed EoE) in patients referred to the ED of the Vienna general hospital (Allgemeines Krankenhaus [AKH] Wien) between 2013 and 2023. The data for the chart review were derived from the patient history in the hospital’s respective computer system (AKIM). A search for “bolus impaction, food impaction, impaction” was done and patients with an EFI needing an esophagogastroduodenoscopy (EGD) were included. The suspected reason for EFI was derived from the endoscopy report. Additionally, all endoscopy reports including images and patient histories were again reviewed by an experienced physician in EoE (PS) who made a suspected final diagnosis.

A descriptive statistical analysis of the data was performed. Categorical data are presented as raw numbers and percentages. Within the binary logistic regression model, gender (male/female), age (under 50/over 50), year of bolus obstruction, and the suspected EoE diagnosis (yes/no) were used as predictors for variable “biopsy” (yes/no, dependent variable). To test whether there is an association between age (< 50/50 +), gender, year of biopsy, and biopsy (yes/no), a four-field correlation was performed.

The study was approved by the Ethical Committee of the Medical University of Vienna (No 1246/2023).

## Results

**Fig. 1:**
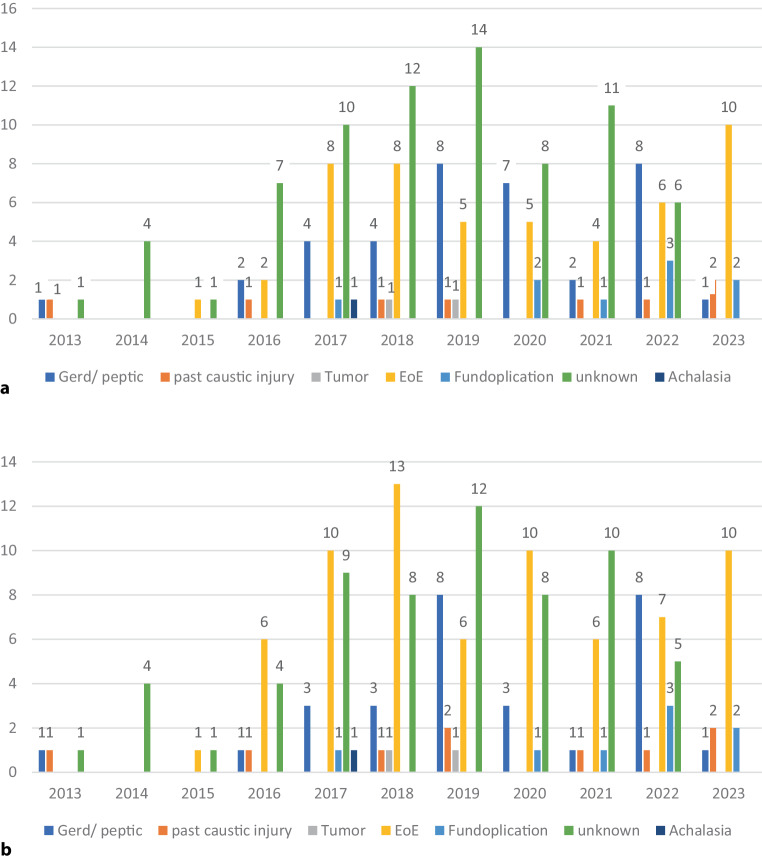
Suspected diagnosis by **a** endoscopist and **b** after review by expert

Between 2013 and 2023, 180 cases of EFI (median age 57 years, 67% male) presented at the ED and received an EGD (Table 1). Overall, the most common suspected diagnosis by the endoscopists were “unknown” (*n* = 74, 41%), followed by EoE (*n* = 49, 27%), and GERD (*n* = 37, 21%). After review by an expert, the most probable diagnosis was EoE (*n* =69, 38%) followed by an unknown etiology (*n* = 62, 34%) and GERD (*n* = 29, 16%) (Fig. 1).

**Table 1:** Esophageal food impaction (EFI) characteristics

Cases with EFI	(*n* = 180) (%)
Male	122 (67)
Age (median years, IQR)	57 (31)
Previously diagnosed EoE	8 (4)
*Retrieval technique*	
Push	125 (69)
Pull	47 (26)
Unknown	8 (5)
Biopsies	33 (18)
Barium swallow examination before endoscopy	118 (65.6)
Medication at discharge	72 (39)
PPI	68 (37)
Budesonide orodispersible tablet	4 (2)
Follow-up after EFI	109 (60)

*EoE* eosinophilic esophagitis, *PPI* proton pump inhibitor, *EFI* esophageal food impaction

**Fig. 2:**
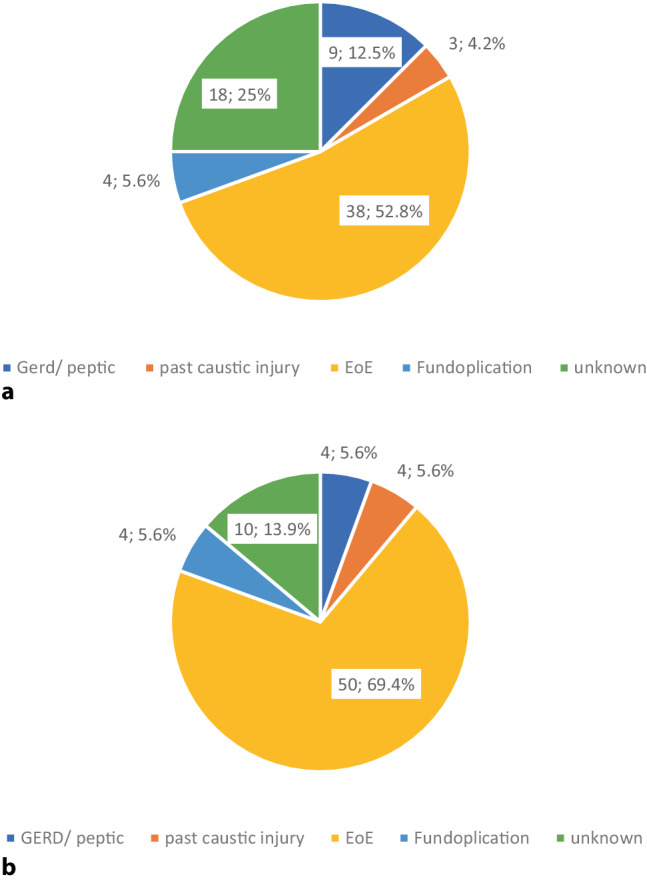
Suspected diagnosis by **a** endoscopist and **b** after review by expert in patients < 50 years of age

**Fig. 3:**
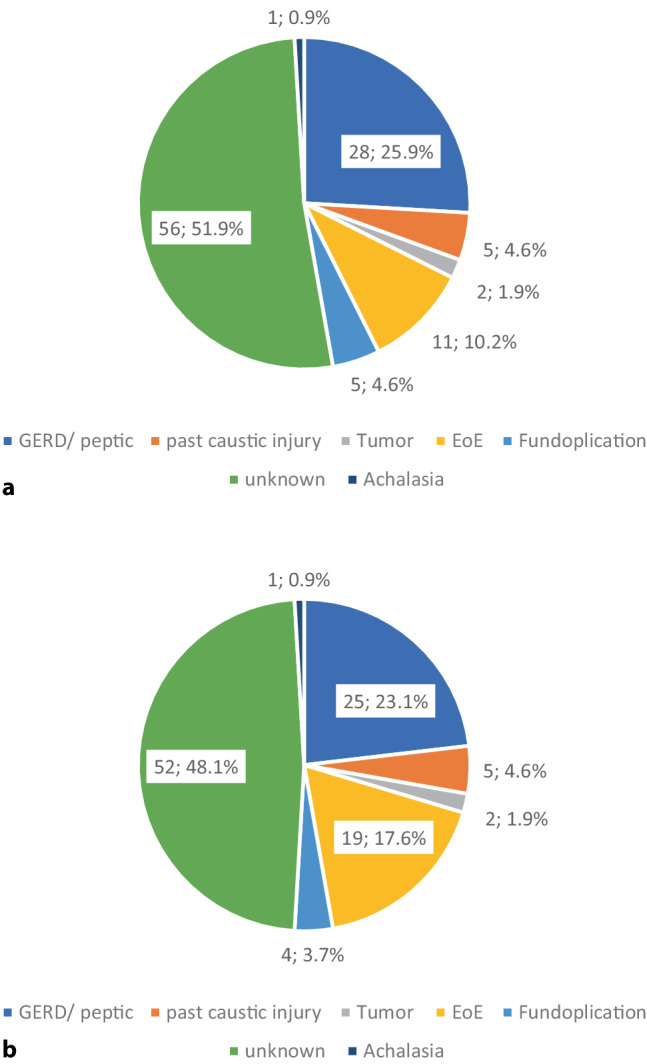
Suspected diagnosis by **a** endoscopist and **b** after review by expert in patients ≥ 50 years of age

In patients < 50 years of age (*n* = 72), the main suspected diagnosis was EoE, followed by an unknown etiology and GERD (Fig. 2). In people ≥ 50 years of age (*n* = 108), the etiology remained unknown in half of patients, followed by GERD (*n* = 25, 23%) and EoE (*n* = 19, 18%) (Fig. 3).

**Fig. 4:**
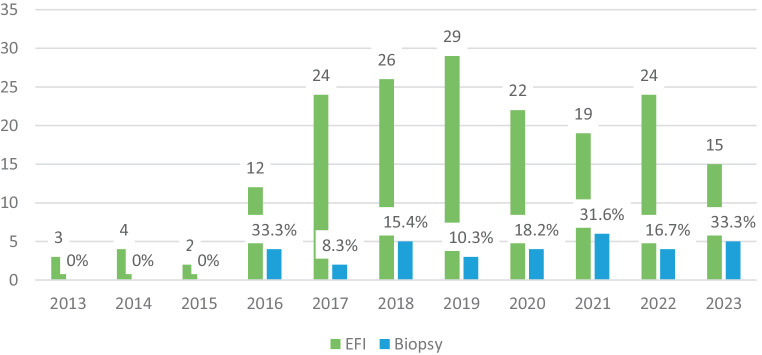
Esophageal food impactions (EFI) and biopsy rate from 2013–2023

From 2013–2023, biopsy was taken in 18% of EFI cases without an increase in recent years (Fig. 4). However, when a diagnosis of EoE was suspected, a biopsy was obtained in 48%.

In only 7.7% (*n* = 14) of all EFI independently of age, EoE diagnosis was histologically confirmed at the time of index endoscopy.

Overall, “Suspicion of EoE by the endoscopist” was the only significant variable associated with the decision to perform a biopsy in the emergency setting of an EFI (EoE suspicion Exp (B) = 4.03, *p* = 0.004). In this case the probability of receiving a biopsy increased by a factor of 4.03. The variables “age” (Exp (B) = 0.74, *p* = 0.535), “gender” (Exp (B) = 9.54, *p* = 0.158) and “year of bolus obstruction” (Exp (B) = 0.884, *p* = 0.172) were not significant.

## Discussion

Esophageal food impaction (EFI) is an increasing gastrointestinal emergency that parallels the rise of EoE [11]. Current guidelines highlight the need for esophageal biopsies in case of EFI to make a proper and timely diagnosis. However, our main findings conclude (1) in case of emergency EGD due to EFI, biopsy rate is rather low with only 18%, which has not increased over the last 10 years; (2) in many patients the etiology of EFI remains unclear and the most common suspected diagnosis is EoE; (3) although the best predictor for obtaining biopsies is “suspected EoE” in the endoscopy report, only in 48% of all suspected EoE patients were biopsies taken.

Similar to a recently published paper by Lee et al. [12], only in a minority of patients with EFI biopsies were performed. There are many potential reasons for this low rate. In addition of the unawareness of the endoscopist, no availability of an endoscopy nurse at off-hour or fear of complications [13] could be an explanation. Although recently published guidelines recommend taking biopsies at the index endoscopy in case of EFI [14], most endoscopists are unaware of the importance of histological work-up in case of EFI [6, 15] and start an empiric therapy with proton pump inhibitors that may even mask EoE in a follow-up endoscopy [16]. Since many EFI occur after regular working hours and an endoscopy nurse or assistant is not available at all time, endoscopists must obtain and process biopsies on their own. In many centers, the access to endoscopy assistance in the evening and at night [17] is similar to our institution making it likely that similar hurdles exist. Furthermore, EoE is a patchy disease and at least 6 biopsies should be obtained to optimize the possibility of obtaining a positive diagnosis of EoE [18]. Even in case the on-call endoscopist is aware of the importance of a histological work-up, some endoscopists fear complications and only take an insufficient number of biopsies [19]. Our data thus support the results of a Danish study which showed that two-thirds of patients never received a diagnosis after an EFI [20] and is in line with a Polish study that showed that over a 5 year period only 33% of patients had diagnostic investigations performed after EFI [21].

Although we do not have evidence that untreated EoE results in higher mortality, the incidence of esophageal strictures rises significantly in patients with a longer diagnostic delay [22]. Therefore, early diagnosis is important to prevent fibrosis [22], improve quality of life [23], and minimize the risk of a further EFI [24]. In line with many other studies [11, 25, 26], we could demonstrate that EoE is the main cause of EFI in young patients. Since young people are often unwilling to see a doctor, it can be hypothesized that EFI represents an “ideal event” to diagnose EoE in this population.

Our single center, retrospective study has some limitations. We could only draw the suspected diagnosis from the endoscopy report. However, almost all endoscopy reports had images included and a medical history was taken at the emergency report. An EoE-experienced physician (PS) went through each endoscopy and medical history and made a suspected diagnosis. Unfortunately, our endoscopy and histology reports do not mention how many biopsies were taken. It therefore remains unclear in how many cases a sufficient number of biopsies were actually taken. If the correct number of biopsies had been taken in all cases, it could be assumed that the prevalence of EoE is even higher.

In summary, although EoE is the most probable cause in the majority of EFI, biopsy rate is rather low. There should be an improvement in the management of patients with EFI to increase biopsy rates and to reduce the rate of undiagnosed EoE. We advocate to take biopsies in all patients with EFI even in case of a macroscopically normal appearing esophagus.

## References

[1] Hahn JW, Lee K, Shin JI, Cho SH, Turner S, Shin JU, et al. Global incidence and prevalence of eosinophilic esophagitis, 1976–2022: a systematic review and meta-analysis. Clin Gastroenterol Hepatol. 2023;21(13):3270–3284.e77. 10.1016/j.cgh.2023.06.005. 37331411 10.1016/j.cgh.2023.06.005

[2] de Rooij WE, Barendsen ME, Warners MJ, van Rhijn BD, Verheij J, Bruggink AH, Bredenoord AJ. Emerging incidence trends of eosinophilic esophagitis over 25 years: results of a nationwide register-based pathology cohort. Neurogastroenterol Motil. 2021;33(7):e14072. 10.1111/nmo.14072. 33426755 10.1111/nmo.14072 PMC8365671

[3] Biedermann L, Straumann A, Greuter T, Schreiner P. Eosinophilic esophagitis—established facts and new horizons. Semin Immunopathol. 2021;43(3):319–35. 10.1007/s00281-021-00855-y. 34097125 10.1007/s00281-021-00855-y PMC8241662

[4] Gómez-Aldana A, Jaramillo-Santos M, Delgado A, Jaramillo C, Lúquez-Mindiola A. Eosinophilic esophagitis: current concepts in diagnosis and treatment. World J Gastroenterol. 2019;25(32):4598–613. 10.3748/wjg.v25.i32.4598. 31528089 10.3748/wjg.v25.i32.4598 PMC6718043

[5] Lam AY, Lee JK, Coward S, Kaplan GG, Dellon ES, Bredenoord AJ, et al. Epidemiologic burden and projections for eosinophilic esophagitis-associated emergency department visits in the United States: 2009–2030. Clin Gastroenterol Hepatol. 2023;21(12):3041–3050.e3. 10.1016/j.cgh.2023.04.028. 37164113 10.1016/j.cgh.2023.04.028

[6] Schreiner P, Safroneeva E, Schoepfer A, Greuter T, Biedermann L, Schlag C, et al. Management of eosinophilic esophagitis associated food impaction in Europe and the United States. Dis Esophagus. 2022;35(9):doac3. 10.1093/dote/doac003. 35088073 10.1093/dote/doac003

[7] Murray FR, Kreienbühl A, Straumann A, Biedermann L, Schreiner P. Natural history of patients lost to follow-up after esophageal food impaction. Clin Gastroenterol Hepatol. 2023;21(9):2413–4. 10.1016/j.cgh.2022.07.007. 35868442 10.1016/j.cgh.2022.07.007

[8] Chang JW, Olson S, Kim JY, Dolan R, Greenson J, Sanders G, Rubenstein JH. Loss to follow-up after food impaction among patients with and without eosinophilic esophagitis. Dis Esophagus. 2019;32(12):doz56. 10.1093/dote/doz056. 31175359 10.1093/dote/doz056 PMC9115375

[9] Murray FR, Kreienbuehl AS, Greuter T, Nennstiel S, Safroneeva E, Saner C, et al. Diagnostic delay in patients with eosinophilic esophagitis has not changed since the first description 30 years ago: diagnostic delay in eosinophilic esophagitis. Am J Gastroenterol. 2022;117(11):1772–9. 10.14309/ajg.0000000000001950. 35971224 10.14309/ajg.0000000000001950

[10] Guo H, Hamilton P, Enns E, Gupta M, Andrews CN, Nasser Y, et al. Postendoscopy care for patients presenting with esophageal food bolus impaction: a population-based multicenter cohort study. Am J Gastroenterol. 2023;118(10):1787–96. 10.14309/ajg.0000000000002392. 37410911 10.14309/ajg.0000000000002392

[11] Lenz CJ, Leggett C, Katzka DA, Larson JJ, Enders FT, Alexander JA. Food impaction: etiology over 35 years and association with eosinophilic esophagitis. Dis Esophagus. 2018;32(4):doy93. 10.1093/dote/doy093. 10.1093/dote/doy093 PMC6437263 30295715

[12] Lee C, Sievers TJ, Vaughn BP. Diagnosis of eosinophilic esophagitis at the time of esophageal food impaction. J Clin Med. 2023;12(11):3768. 10.3390/jcm12113768. 37297963 10.3390/jcm12113768 PMC10253473

[13] Sengupta N, Tapper EB, Corban C, Sommers T, Leffler DA, Lembo AJ. The clinical predictors of aetiology and complications among 173 patients presenting to the emergency department with oesophageal food bolus impaction from 2004–2014. Aliment Pharmacol Ther. 2015;42(1):91–8. 10.1111/apt.13237. 25963885 10.1111/apt.13237

[14] Dhar A, Haboubi HN, Attwood SE, Auth MKH, Dunn JM, Sweis R, et al. British Society of Gastroenterology (BSG) and British Society of Paediatric Gastroenterology, Hepatology and Nutrition (BSPGHAN) joint consensus guidelines on the diagnosis and management of eosinophilic oesophagitis in children and adults. Gut. 2022;71(8):1459–87. 10.1136/gutjnl-2022-327326. 35606089 10.1136/gutjnl-2022-327326 PMC9279848

[15] Schreiner P, Balcar L, Schlager H, Madl C, Ziachehabi A, Mader M, et al. Management of suspected and known eosinophilic esophagitis‑a nationwide survey in Austria. Wien Klin Wochenschr. 2023;135(15–16):406–13. 10.1007/s00508-023-02198-0. 37071203 10.1007/s00508-023-02198-0 PMC10444684

[16] Hillman L, Donohue S, Broman AT, Hoversten P, Gaumnitz E, Lomeli L. Empiric proton pump inhibitor therapy after esophageal food impaction may mask eosinophilic esophagitis diagnosis at follow-up. Dis Esophagus. 2021;34(11):doab30. 10.1093/dote/doab030. 33987650 10.1093/dote/doab030

[17] Muthiah KC, Enns R, Armstrong D, Noble A, Gray J, Sinclair P, et al. A survey of the practice of after-hours and emergency endoscopy in Canada. Can J Gastroenterol. 2012;26(12):871–6. 10.1155/2012/951071. 23248785 10.1155/2012/951071 PMC3551559

[18] Nielsen JA, Lager DJ, Lewin M, Rendon G, Roberts CA. The optimal number of biopsy fragments to establish a morphologic diagnosis of eosinophilic esophagitis. Am J Gastroenterol. 2014;109(4):515–20. 10.1038/ajg.2013.463. 24445569 10.1038/ajg.2013.463

[19] Schreiner P, Balcar L, Schlager H, Madl C, Ziachehabi A, Mader M, et al. Management of suspected and known eosinophilic esophagitis—a nationwide survey in Austria. Wien Klin Wochenschr. 2023;135(15–16):406–13. 10.1007/s00508-023-02198-0. 37071203 10.1007/s00508-023-02198-0 PMC10444684

[20] Terkelsen JH, Hollænder M, Bredal K, Nielsen SM, Thomsen KVK, Baggerman A, et al. A retrospective cohort study on oesophageal food bolus obstruction in the North Denmark region in 2021—two thirds were never diagnosed with a cause. BMC Gastroenterol. 2024; 10.1186/s12876-023-03077-8. 38166672 10.1186/s12876-023-03077-8 PMC10759704

[21] Arman S, Vijendren A, Lyons M. Outcome and follow-up of patients requiring emergency oesophagoscopy for food bolus obstruction over a 5‑year period. Pol J Otolaryngol. 2020;74(3):29–32. 10.5604/01.3001.0013.5261. 10.5604/01.3001.0013.5261 32398386

[22] Schoepfer AM, Safroneeva E, Bussmann C, Kuchen T, Portmann S, Simon HU, Straumann A. Delay in diagnosis of eosinophilic esophagitis increases risk for stricture formation in a time-dependent manner. Gastroenterology. 2013;145(6):1230–1236.e1‑2. 10.1053/j.gastro.2013.08.015. 23954315 10.1053/j.gastro.2013.08.015

[23] Mukkada V, Falk GW, Eichinger CS, King D, Todorova L, Shaheen NJ. Health-related quality of life and costs associated with eosinophilic esophagitis: a systematic review. Clin Gastroenterol Hepatol. 2018;16(4):495–503.e8. 10.1016/j.cgh.2017.06.036. 28655543 10.1016/j.cgh.2017.06.036

[24] Kuchen T, Straumann A, Safroneeva E, Romero Y, Bussmann C, Vavricka S, et al. Swallowed topical corticosteroids reduce the risk for long-lasting bolus impactions in eosinophilic esophagitis. Allergy. 2014;69(9):1248–54. 10.1111/all.12455. 24894658 10.1111/all.12455

[25] Longstreth GF, Longstreth KJ, Yao JF. Esophageal food impaction: epidemiology and therapy. A retrospective, observational study. Gastrointest Endosc. 2001;53(2):193–8. 10.1067/mge.2001.112709. 11174291 10.1067/mge.2001.112709

[26] Melendez-Rosado J, Corral JE, Patel S, Badillo RJ, Francis D. Esophageal food impaction: causes, elective intubation, and associated adverse events. J Clin Gastroenterol. 2019;53(3):179–83. 10.1097/MCG.0000000000001004. 29517706 10.1097/MCG.0000000000001004

